# Analysis of Serial Isolates of *mcr-1*-Positive Escherichia coli Reveals a Highly Active IS*Apl1* Transposon

**DOI:** 10.1128/AAC.00056-17

**Published:** 2017-04-24

**Authors:** Erik Snesrud, Ana C. Ong, Brendan Corey, Yoon I. Kwak, Robert Clifford, Todd Gleeson, Shannon Wood, Timothy J. Whitman, Emil P. Lesho, Mary Hinkle, Patrick McGann

**Affiliations:** a Multidrug-Resistant Organism Repository and Surveillance Network, Walter Reed Army Institute of Research, Silver Spring, Maryland, USA; b Department of Infectious Diseases, Walter Reed National Military Medical Center, Bethesda, Maryland, USA

**Keywords:** colistin, *mcr-1*, plasmids

## Abstract

The emergence of a transferable colistin resistance gene (*mcr-1*) is of global concern. The insertion sequence IS*Apl1* is a key component in the mobilization of this gene, but its role remains poorly understood. Six Escherichia coli isolates were cultured from the same patient over the course of 1 month in Germany and the United States after a brief hospitalization in Bahrain for an unconnected illness. Four carried *mcr-1* as determined by real-time PCR, but two were negative. Two additional *mcr-1*-negative E. coli isolates were collected during follow-up surveillance 9 months later. All isolates were analyzed by whole-genome sequencing (WGS). WGS revealed that the six initial isolates were composed of two distinct strains: an initial ST-617 E. coli strain harboring *mcr-1* and a second, unrelated, *mcr-1*-negative ST-32 E. coli strain that emerged 2 weeks after hospitalization. Follow-up swabs taken 9 months later were negative for the ST-617 strain, but the *mcr-1*-negative ST-32 strain was still present. *mcr-1* was associated with a single copy of IS*Apl1*, located on a 64.5-kb IncI2 plasmid that shared >95% homology with other *mcr-1* IncI2 plasmids. IS*Apl1* copy numbers ranged from 2 for the first isolate to 6 for the final isolate, but IS*Apl1* movement was independent of *mcr-1*. Some movement was accompanied by gene disruption, including the loss of genes encoding proteins involved in stress responses, arginine catabolism, and l-arabinose utilization. These data represent the first comprehensive analysis of IS*Apl1* movement in serial clinical isolates and reveal that, under certain conditions, IS*Apl1* is a highly active IS element whose movement may be detrimental to the host cell.

## INTRODUCTION

Colistin (polymyxin E) has reemerged in the last decade as a “last-line” antibiotic treatment for serious infections caused by carbapenem-resistant organisms (CROs) (1). A major reason for the renewal of interest in this drug was the belief that colistin resistance developed only through point mutation and was therefore not transmitted horizontally (2). This paradigm was recently upended with the discovery of the transferable colistin resistance gene *mcr-1* (3). Remarkably, this gene appears to have been circulating undetected for at least 3 decades (4).

The ubiquitous distribution of *mcr-1* is now accepted. Notably, the gene has been identified in isolates from animal sources at a much higher frequency than that for human isolates, and along with other lines of evidence, these data suggest that the reservoir for *mcr-1* is in animals (5). While animals and animal products are key vectors in the spread of *mcr-1*, colonization of humans with bacterial strains carrying this gene has been reported among travelers to the Caribbean, China, Southeast Asia, and southern Africa (6–9). However, other reported cases of humans colonized with *mcr-1*-containing bacteria have no obvious route of transmission and lack any exposure to colistin (10, 11).

The dissemination of *mcr-1* has been facilitated greatly by its location on a wide variety of plasmids, including many plasmid replicons associated with antibiotic resistance gene spread, such as IncF, IncH12, IncI2, IncP, and IncX4 (see reference 12 for a comprehensive list). In many cases, the presence of *mcr-1* is intimately associated with the transposon IS*Apl1*. A recent analysis of *mcr-1*-containing isolates suggests that the gene was initially mobilized by a composite transposon composed of two directly orientated copies of IS*Apl1* that subsequently lost one or both copies of the transposon, most likely through illegitimate recombination (12). This loss may have served to stabilize the gene within the plasmid vector, thus facilitating its dissemination (12).

IS*Apl1* was first detected in Actinobacillus pleuropneumoniae, a member of the Pasteurellaceae family that causes fibrinous and necrotic pleuropneumonia in pigs (13). It belongs to the IS*30* family of transposons and is flanked by 27-bp inverted repeats with six mismatches (designated inverted repeat left and inverted repeat right [IRL and IRR, respectively]). IS*Apl1* contains a 927-bp open reading frame (ORF) that encodes a DD(E/D) superfamily transposase protein that generates 2-bp target site duplications (TSDs) upon integration (13). Like other members of the IS*30* family, IS*Apl1* is typically present in multiple copies in the genome, and these insertion sites are notable for their high AT content (12, 14). The insertions appear to remain stable for at least 3 weeks during passage of A. pleuropneumoniae in animals, but this has been demonstrated only for monomeric forms of IS*Apl1* (13). However, it remains unclear how active the transposition of IS*Apl1* is within a cell.

In this study, whole-genome sequencing (WGS) was used to analyze four serial isolates of an *mcr-1*-containing Escherichia coli strain obtained from the same patient over the course of a month. The data revealed that the number of IS*Apl1* copies varied from 2 to 6 across the four isolates, but IS*Apl1* movement was independent of *mcr-1*. Notably, a second, unrelated E. coli strain lacking *mcr-1* was also isolated after 3 weeks. Upon discharge of the patient, rectal swabs from the patient were negative for E. coli, though no antibiotics were administered during this period. Two follow-up swabs 9 and 10 months later revealed that the second E. coli strain was still present, but *mcr-1* could not be detected using real-time PCR (RT-PCR), and no growth was observed on agar supplemented with colistin.

In late 2015, a middle-aged male was transferred to a U.S. military hospital in Germany after a 3-week hospitalization in Bahrain, where he had received empirical ceftriaxone for “fevers.” Other medical, travel, exposure, and treatment histories were unobtainable. Admission perirectal surveillance cultures in Germany grew extended-spectrum β-lactamase (ESBL)-producing E. coli (MRSN 352231), and contact precautions were initiated. Five days later, the patient was transferred to the Walter Reed National Military Medical Center (WRNMMC), where contact precautions were continued per infection prevention and control policy for all medically evacuated (medevaced) patients from overseas. Follow-up groin and perirectal surveillance swabs during hospitalization at WRNMMC grew ESBL-producing E. coli strains with two different morphologies. The final swab prior to discharge was negative for ESBL-producing E. coli. No ESBL-producing E. coli strains were isolated from urine and throat cultures throughout the period. The patient received no antibiotics during his hospitalization.

Retrospective screening for *mcr-1* (11) identified *mcr-1* in four of the six isolates cultured during hospitalization. Follow-up perirectal surveillance swabs in July and August 2016 showed no growth on colistin-impregnated Mueller-Hinton agar plates, and the swabs were negative by real-time PCR for the presence of *mcr-1*. Notably, an ESBL-producing *mcr-1*-negative E. coli strain that had the same antibiotic susceptibility profile as the *mcr-1*-negative isolates obtained 9 months prior was isolated.

## RESULTS

### Phenotypic and clinical characteristics of isolates.

Six E. coli isolates, four carrying *mcr-1*, were collected during hospitalization from groin or perirectal surveillance swabs obtained over the course of 1 month. Two additional *mcr-1*-negative isolates were collected from perirectal surveillance swabs obtained 9 months later. The first isolate (MRSN 352231; *mcr-1* positive) was cultured from a perirectal surveillance swab in Germany on day 1. In addition to being colistin resistant, the isolate was resistant to a range of antibiotics, including 3rd- and 4th-generation cephalosporins, ciprofloxacin, and levofloxacin, but was sensitive to the carbapenems and aminoglycosides (Table 1). MRSN 346355 (*mcr-1* positive) was cultured from a groin surveillance swab on day 6, after the patient had been repatriated to the United States, and it displayed the same antibiotic resistance profile as MRSN 352231. The patient received definitive care but no antibiotics over the following weeks, and on day 26, a follow-up groin surveillance swab isolated MRSN 346595 (*mcr-1* positive) and MRSN 346647 (*mcr-1* negative), two E. coli strains with distinct morphologies. MRSN 346595 had the same antibiotic susceptibility profile as the two previous isolates, but MRSN 346647 was sensitive to the fluoroquinolones and nitrofurantoin (Table 1). A perirectal surveillance swab taken on day 30 also isolated two distinct E. coli isolates (MRSN 346638 and MRSN 346629) with the same susceptibility profiles as MRSN 346595 and MRSN 346647, respectively (Table 1).

**TABLE 1:** Primary characteristics of E. coli strains used for this study

Strain	ST determined by MLST^*b*^	Day after initial patient hospitalization	Source^*c*^	Presence of *mcr-1*^*d*^	MIC (μg/ml)^*a*^			
					CST	CIP	LVX	NIT
MRSN 352231	617	1	Perirectal	+	4	>2	>4	32
MRSN 346355	617	6	Groin	+	4	>2	>4	32
MRSN 346595	617	26	Groin	+	4	>2	>4	32
MRSN 346647	32	26	Groin	−	**≤0.5**	**1**	**≤1**	**≤16**
MRSN 346638	617	30	Perirectal	+	4	>2	>4	32
MRSN 346629	32	30	Perirectal	−	**≤0.5**	**1**	**≤1**	**≤16**
MRSN 418111	32	258	Perirectal	−	**≤0.5**	**1**	**≤1**	**≤16**
MRSN 418944	32	291	Perirectal	−	**≤0.5**	**1**	**≤1**	**≤16**

a Abbreviations: CST, colistin; CIP, ciprofloxacin; LVX, levofloxacin; NIT, nitrofurantoin. All isolates were resistant to amoxicillin-clavulanic acid, aztreonam, cefepime, ceftazidime, ertapenem, imipenem, tetracycline, and trimethoprim-sulfamethoxazole. All isolates were sensitive to the aminoglycosides amikacin, gentamicin, and tobramycin. Differences in antibiotic susceptibilities in the ST-32 isolates compared to the ST-617 isolates are highlighted in bold.

b *In silico* multilocus sequence type based on the MLST scheme developed by the University of Warwick, United Kingdom (http://mlst.warwick.ac.uk/mlst/dbs/Ecoli).

c Clinical sites where surveillance swabs were used to culture the isolate.

d Presence (+) or absence (−) of *mcr-1*, based on real-time PCR and confirmed by sequencing.

In May 2016, the Multidrug-Resistant Organism Repository and Surveillance Network (MRSN) commenced retroactive testing of all Enterobacteriaceae for *mcr-1* by use of real-time PCR. In July, MRSN 352231, 346355, 346595, and 346638 tested positive for the gene, but MRSN 346647 and 346629 were negative. Colistin Etest and manual broth microdilution confirmed that the four isolates carrying *mcr-1* had a colistin MIC of 4 μg/ml, whereas the two *mcr-1*-negative isolates had colistin MICs of ≤0.5 μg/ml (Table 1). The relevant public health authorities were contacted, and follow-up perirectal surveillance swabs were performed on days 258 and 291. A single E. coli strain was isolated from each swab (MRSN 41811 and MRSN 418944, respectively), but the isolates had colistin MICs of <0.25 μg/ml and were negative for *mcr-1* by real-time PCR. Notably, the isolates had the same susceptibility profile as the two previous *mcr-1*-negative isolates collected during the initial hospitalization (MRSN 346647 and 346629).

### Whole-genome sequencing of clinical isolates.

Short- and long-read sequencing was performed on all eight E. coli strains. *In silico* multilocus sequence typing (MLST) assigned the four *mcr-1*-positive isolates to ST-617, a member of the ST-10 clonal complex belonging to phylogenetic group A, which is associated with commensal strains. A BLAST comparison with other E. coli sequences in GenBank indicated that the isolates shared >99% homology across ∼3.3 Mb of the 4.79-Mb genome with E. coli Sanji (accession no. CP011061, submitted March 2015), isolated from the duodenum of a pheasant in China. In contrast, the four *mcr-1*-negative isolates were assigned to ST-32, a member of clonal complex 32 (CC32) that spans phylogenetic groups A, B, and D. Further *in silico* PCR analysis indicated that these ST-32 isolates belonged to phylogenetic group D (*chuA* and TSPE4.C2 positive and *yjaA* negative) (15), which is associated with virulent extraintestinal strains (16, 17). Whole-genome single nucleotide polymorphism (SNP)-based analysis revealed that the ST-32 isolates MRSN 346647 and 346629 were identical and were separated from the other two ST-32 isolates (MRSN 41811 and MRSN 418944) by 3 SNPs. Notably, IS*Apl1* was absent from the ST-32 isolates. Similarly, the four ST-617 isolates were genetically identical, with the exception of the IS*Apl1* copy number (see below) and a single nonsynonymous mutation in *ydhJ* that was present in every isolate except the first (MRSN 352231). This mutation results in the replacement of a tryptophan with a glycine (W176G) in the putative membrane fusion protein YdhJ, a component of the YdhJK efflux pump.

WGS data detected 8 antibiotic resistance genes in the ST-32 isolates and 13 in the ST-617 isolates, which correlated strongly with phenotype (Table 2). Both groups of isolates carried the ESBL gene *bla*_CTX-M-15_, though the gene was carried chromosomally in the ST-617 isolates and on an IncF1B plasmid in the ST-32 isolates (Table 2). Both groups also shared the penicillinase gene *bla*_TEM-1B_ and the tetracycline resistance gene *tet*(*A*). In addition, the ST-617 isolates carried genes encoding resistance to colistin (*mcr-1*) and carbenicillin (*bla*_PSE-1_ [same as *bla*_CARB-2_]). Notably, the ST-617 isolates were classified as resistant to the fluoroquinolones based on CLSI guidelines, but they lacked any fluoroquinolone resistance genes. However, an analysis of the *gyrA*, *parC*, and *parE* genes detected nonsynonymous mutations in GyrA (S83L and D87N), ParC (S80I), and ParE (S458A) that have previously been shown to confer high-level fluoroquinolone resistance (18). In contrast, the ST-32 isolates were sensitive to the fluoroquinolones, though the MIC of ciprofloxacin was 1 μg/ml. This correlates with the presence in these isolates of *qnrS1*, which confers low-level fluoroquinolone resistance (19). Unlike the ST-617 isolates, the ST-32 isolates showed no mutations in the *gyrA*, *parC*, or *parE* gene that are known to be involved in fluoroquinolone resistance. However, the ST-32 isolates had nonsynonymous mutations in the MexR (K62R, G103S, and Y137H) and SoxR (T38S and G74R) regulators. Mutations in these regulators were recently linked to increased low-level fluoroquinolone resistance in strains expressing Qnr (20).

**TABLE 2:** Antibiotic resistance genes carried by E. coli isolates

Isolate group and gene^*a*^	Location of gene (Inc group, size [kb])^*b*^	Resistance^*c*^
ST-32 isolates		
*bla*_CTX-M-15_	Plasmid (F1B, 113.1)	β-Lactams (extended spectrum)
*bla*_TEM-1B_	Plasmid (F1B, 113.1)	β-Lactams (penicillin)
*aph*(*6*)-*Ia* (*strA*)	Plasmid (F1B, 113.1)	Aminoglycosides (streptomycin)
*aph*(*6*)-*Id* (*strB*)	Plasmid (F1B, 113.1)	Aminoglycosides (streptomycin)
*qnrS1*	Plasmid (F1B, 113.1)	Quinolones
*sul2*	Plasmid (F1B, 113.1)	Sulfonamides
*tet*(*A*)	Plasmid (F1B, 113.1)	Tetracyclines
*dfrA14*	Plasmid (F1B, 113.1)	Trimethoprim
ST-617 isolates		
*ant*(*3*″)-*Ia* (*aadA1*)	Plasmid (R, 67.9)	Aminoglycosides (streptomycin)
*ant*(*3*″)-*Ia* (*aadA2*)	Plasmid (R, 67.9)	Aminoglycosides (streptomycin)
*bla*_CTX-M-15_	Chromosome	β-Lactams (extended spectrum)
*bla*_TEM-1B_	Plasmid (I1, 102.3–208)	β-Lactams (penicillin)
*bla*_PSE-1_ (*bla*_CARB-2_)	Plasmid (R, 67.9)	β-Lactams (penicillin and carbenicillin)
*erm*(*42*)	Plasmid (I1, 102.3–208)	Macrolides
***mcr-1***	**Plasmid (I2, 64.5)**	**Polymyxins**
*cmlA1*	Plasmid (R, 67.9)	Phenicols
*floR*	Plasmid (I1, 102.3–208)	Phenicols
*sul3*	Plasmid (R, 67.9)	Sulfonamides
*tet*(*A*)	Plasmid (R, 67.9)	Tetracyclines
*dfrA16*	Plasmid (R, 67.9)	Trimethoprim

a Based on the closest match obtained using the BLAST algorithm (http://blast.ncbi.nlm.nih.gov). The *mcr-1* data discussed in the present study are shown in bold.

b Based on long-read sequence analysis. The plasmid incompatibility group and approximate size are provided where relevant.

c Predicted resistance phenotype. Note that for clarity, where detailed, only clinically relevant antibiotics are listed.

Two further features of the whole-genome sequencing analysis were notable. First, a combination of long- and short-read sequencing identified a stretch of 35 tandem duplications of *bla*_TEM-1B_ flanked by IS*26* in the IncI1 plasmid in MRSN 352231 only, resulting in the plasmid increasing in size from 102.3 kb to 208 kb in this isolate (Table 2). Studies are ongoing to investigate this structure further. Second, genomic analysis revealed the presence of 28 different insertion sequence (IS) elements located in 81 different positions throughout the chromosome and plasmids (see Table S1 in the supplemental material). One of these was a novel IS*5*-like element that was found in all ST-617 isolates. It has 80.7% identity to the canonical IS*5* sequence and, like other members of the IS*5* family, generates 4-bp target site duplications (TSD) upon integration. The new sequence was designated IS*Ec68*.

### Plasmid analysis by long-read sequencing.

Long-read sequencing was performed on all four ST-617 isolates and a single ST-32 isolate (MRSN 346647). The ST-617 strains assembled into a single chromosomal contig and five plasmids, while the ST-32 isolates assembled into a single chromosomal contig and two plasmids (Table 2). In addition to three plasmids carrying a variety of antibiotic resistance genes, all ST-617 isolates carried a 61.1-kb IncFII plasmid with no antibiotic resistance genes and a small, 5.3-kb col*RNA1* plasmid. Similarly, all ST-32 isolates carried a small, 5.2-kb col*8282* plasmid (data not shown). With the exception of the chromosomally carried *bla*_CTX-M-15_ gene in the ST-617 isolates, all antibiotic resistance genes were plasmid borne, highlighting the critical role that these structures play in the dissemination of antibiotic resistance.

### Analysis of *mcr-1*-containing plasmid pMR0716_mcr1.

**FIG 1:**
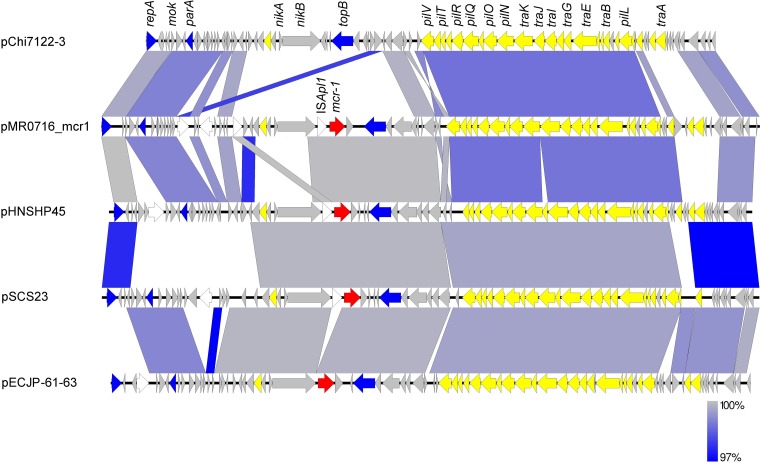
Alignment of selected IncI2 plasmids with pMR0716_mcr. Block arrows indicate confirmed or putative open reading frames (ORFs) and their orientations. Arrow size is proportional to the predicted ORF length. Conjugal transfer genes are indicated by yellow arrows, and *mcr-1* is indicated by a red arrow. Plasmid transfer and mobilization genes are indicated by blue arrows. Putative, hypothetical, and unknown genes are indicated by gray arrows. Genes encoding mobile elements are indicated by white arrows. Regions of homology between the plasmids ranging from 97% to 100% are indicated by the graded shaded regions between sequences.

The colistin resistance gene *mcr-1* was carried on a 64.5-kb IncI2 plasmid, here designated pMR0716_mcr1, and was the only antibiotic resistance gene present on this element (Table 2). An alignment with other IncI2 plasmid sequences showed considerable homology to pHNSHP45, the original plasmid carrying *mcr-1* described by Liu and colleagues (3), sharing an average nucleotide identity by BLAST searches (ANIb) of 98.79% (Fig. 1). pMR0716_mcr1 also shares an ANIb of 98.04% with pChi7122-3, a 56.7-kb plasmid that is carried by the avian-pathogenic E. coli (APEC) strain 7122 but lacks the *mcr-1* gene (Fig. 1). Like other IncI2 plasmids, pMR0716_mcr1 is characterized by large numbers of putative and hypothetical genes and genes of unknown function, likely reflecting its association with animal and food sources.

### Genetic context of *mcr-1* and IS*Apl1*.

**FIG 2:**
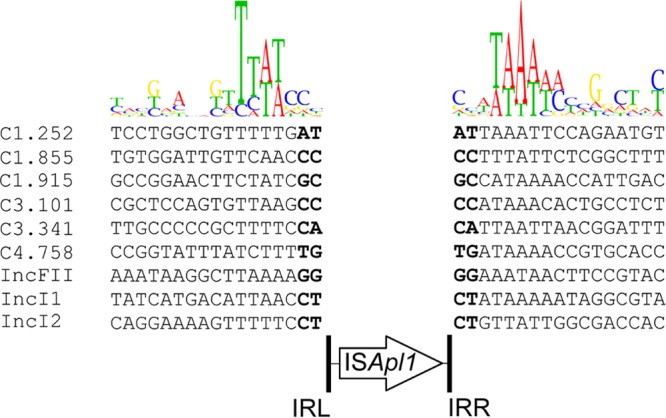
Consensus sequence for all IS*Apl1* genomic insertion sites. The overall height of the stack indicates the sequence conservation at that position, while the height of each symbol within the stack indicates the relative frequency of each nucleic acid at that position. The 2-bp target site duplications generated upon IS*Apl1* insertion are highlighted in bold. Sequence names correspond to the chromosomal (C) or plasmid insertion site, as detailed in Table 3. The consensus alignment was generated using the Geneious R9.1 software suite.

A single copy of *mcr-1* was present on pMR0716_mcr-1 in all ST-617 strains. Similarly to that in many other *mcr-1*-containing plasmids (12), a single copy of IS*Apl1* was located directly upstream of the gene. A second copy of IS*Apl1* was also located on the chromosome of every isolate, but it lacked the attendant *mcr-1* gene where it disrupted the *iraM* gene, which encodes an antiadapter protein that interacts with RssB to prevent the degradation of the stress sigma factor RpoS under magnesium starvation conditions (Table 3). Further analysis of the serial isolates suggested that IS*Apl1* is highly active, with multiple copies inserted throughout the genome. Sequencing of the remaining three *mcr-1*-containing isolates revealed additional copies of IS*Apl1*, again without any attendant *mcr-1* gene, in successive isolates (Table 3). MRSN 346355, collected 6 days after MRSN 352231, contained two additional copies that had inserted into pMR0716_mcr1 and the IncI2 plasmid. In pMR0716_mcr1, IS*Apl1* was located in a noncoding region 167 bp upstream of *bla*_TEM-1b_. The second insertion resulted in disruption of the *ycfA* gene, which encodes the toxin HicA. MRSN 346595, collected on day 26, had three additional copies, one of which was identical to the pMR0716_mcr1 insertion in MRSN 346355. The second copy was inserted into a noncoding region in the IncFII plasmid, 85 bp upstream of a gene encoding a hypothetical protein similar to YigA. The third copy was inserted into the chromosome, where it disrupted a gene encoding a hypothetical protein that is similar to the E. coli inner membrane protein YdiK. The final isolate, MRSN 346638, had four additional copies of IS*Apl1*, all located on the chromosome (Table 3). One insertion disrupted a gene encoding a succinylarginine dihydrolase, while a second disrupted a gene encoding a sugar kinase. The remaining two insertions occurred in noncoding regions 128 bp and 168 bp upstream of genes encoding an ABC transporter substrate-binding protein (NikA-DppA-OppA-like superfamily) and an l-galactonate transporter (MFS superfamily), respectively. An analysis of the genetic environment surrounding the additional copies of IS*Apl1* revealed a significant bias for insertion in AT-rich regions, with a slight central GC bias (Fig. 2), as previously reported for IS*Apl1* (12). Furthermore, each insertion site was flanked by the characteristic 2-bp TSD generated by IS*Apl1* upon insertion (Table 3).

**TABLE 3:** Additional IS*Apl1* insertion sites in clinical isolates

Position^*a*^	TSD^*b*^	Strain(s)	Effect^*c*^
1.252	AT	All isolates	Interruption of *iraM* (encoding an antiadapter protein)
1.855	CC	346595	Interruption of a hypothetical protein gene (encoding an E. coli YdiK-like protein)
1.915	GC	346638	Interruption of an *astB*-like gene (encoding succinylarginine dihydrolase)
3.101	CC	346638	Interruption of an *araB*-like gene (encoding sugar kinase)
3.341	CA	346638	Intergenic
4.758	TG	346638	Intergenic
IncFII	GG	346595	Intergenic
IncI1	AG	346355, 346595	Intergenic
IncI2	AG	346355	Interruption of *ycfA* (encoding HicA toxin)

a Chromosomal (numbers) or plasmid (Inc type) location of IS*Apl1* insertion site. Numbers indicate positions, in megabases, with reference to the origin of chromosomal replication (*oriC*).

b Two-base-pair target site duplication (TSD) flanking the site of IS*Apl1* insertion.

c Predicted phenotypic change resulting from IS*Apl1* insertion at the designated location. “Intergenic” implies insertion in a region with no predicted open reading frames.

## DISCUSSION

Collection of serial *mcr-1*-containing ST-617 E. coli isolates from the same patient over the course of 1 month provided the opportunity to examine the movement of IS*Apl1* during this time. Furthermore, analysis of the serial isolates revealed the emergence of a second, unrelated, *mcr-1*-negative ST-32 E. coli isolate that eventually displaced the original *mcr-1*-containing strain. Long-read whole-genome sequencing analysis of the ST-617 isolates revealed that in successive isolates the number of IS*Apl1* copies throughout the genome consistently increased, from just two in the first isolate collected to six in the final isolate, and that this movement was independent of *mcr-1* (Table 3).

Colonization of travelers by multidrug-resistant strains has long been recognized as an important source for the dissemination of antibiotic resistance genes (21), particularly in areas where these strains are endemic (22). Epidemiological evidence has also indicated that foreign travel is a risk factor for the acquisition of strains carrying *mcr-1*. For example, a recent study of Dutch travelers detected *mcr-1* in fecal samples after travel to Southeast Asia and southern Africa, despite their being negative for the gene prior to travel (9). Similarly, a case of *mcr-1*-positive ST-3944 E. coli isolates cultured from the urine of a patient undergoing a prostate resection was believed to have been acquired in China 2 months prior, where the patient had been hospitalized for acute urinary retention (6). In our study, epidemiological data indicated that the patient most likely acquired the *mcr-1*-carrying E. coli strain in Bahrain, where he had been working extensively for the previous 10 years. This was supported by initial surveillance swabs taken in Germany indicating that the patient was colonized upon admission. Furthermore, a recent study by Sonnevend et al. also showed that *mcr-1* is present in Bahrain, where it was detected in two E. coli isolates cultured from human clinical samples (23). *In silico* MLST assigned the isolate to ST-617, a member of the globally distributed clonal complex 10 (CC10) and part of phylogenetic group A, which is most often associated with commensal E. coli (24). Though the E. coli strains in the study of Sonnevend et al. belonged to different STs (ST-648 and ST-224), both carried *mcr-1* on an IncI2 plasmid (23).

Plasmids represent the primary vehicles for the dissemination of antibiotic resistance genes (25), and *mcr-1* has been found on plasmids representing a diverse range of incompatibility groups. In particular, *mcr-1*-harboring plasmids belonging to the IncI2, IncHI2, and IncX4 families account for the majority of such sequences submitted to GenBank, to date (see reference 12 for a comprehensive list). In this study, *mcr-1* was also carried on an IncI2 plasmid (pMR0716_mcr1) with high homology to other *mcr-1*-containing IncI2 plasmids, in which it was the only antibiotic resistance gene present. However, four additional plasmids were also coresident with pMR0716_mcr1, including an IncR plasmid and an IncI1 plasmid that carried 7 and 3 antibiotic resistance genes, respectively (Table 2). As in pHNSHP45 and other *mcr-1*-containing plasmids, *mcr-1* abutted a single upstream copy of IS*Apl1* and a putative 765-bp downstream ORF encoding a protein similar to a PAP2 superfamily protein. Initial integration of this module into pMR0716_mcr1, most likely due to the movement of an ancestral composite transposon (12), disrupted the *ycfA* gene. This gene encodes HicA, the toxin component of a two-component toxin-antitoxin plasmid addiction module (26, 27) which should no longer be functional in this strain. An analysis of successive isolates indicated that all ST-617 strains, in addition to carrying the original IS*Apl1–mcr-1* module, carried an additional copy of IS*Apl1* in the same location on the chromosome (Table 3). Interestingly, this insertion disrupts the gene encoding IraM, which inhibits RssB activity during magnesium starvation (28). As RssB is involved in the degradation of the stress response regulator RpoB, RpoS degradation in these strains should continue under magnesium-limiting conditions, limiting the bacterial stress response under these conditions (29).

An analysis of successive isolates revealed an additional one to four copies of IS*Apl1* present in the plasmids and chromosome, with the number of additional IS*Apl1* copies increasing over time (Table 3). Notably, IS*Apl1* movement was not accompanied by *mcr-1* movement, providing further evidence that *mcr-1* is mobilized only as part of an IS*Apl1* composite transposon (12). Notably, the insertion sites for the additional copies varied considerably between isolates, with only MRSN 346355 and MRSN 346595 sharing the same site for one of the copies (within the IncI1 plasmid). Four of the insertions resulted in gene disruption, including disruption of the genes encoding a succinylarginine dihydrolase and a sugar kinase, which would prevent arginine catabolism and l-arabinose utilization, respectively. Because IS*Apl1* moves through a replicative mechanism known as copy out-paste in, it leaves a copy of the transposon in the original location during movement (30). Hence, if these isolates truly represented a gradual accumulation of additional IS*Apl1* copies over time, one would expect to see successive isolates sharing the same insertion sites, with additional insertions expanding on these. A plausible explanation is that the four sequenced isolates may represent just a snapshot of an overall population wherein IS*Apl1* is highly active and transposing at a very high frequency. This would result in multiple subpopulations within each isolate that are differentiated by the number and location of IS*Apl1* copies. Functional experiments, including long-read sequencing of multiple colonies from the same culture, should further clarify the nature of the *mcr-1* mobile element context and its origins.

IS movement can have significant impacts on genomic structure and function, many of which can be detrimental to the cell (31). Environmental challenges and stresses can result in a transposition burst, characterized by a sudden increase in IS transposition that can result in significant gene disruption (32). For example, insertion sequence-mediated genomic rearrangements in Burkholderia cenocepacia following oxidative stress altered the pulsed-field gel electrophoresis macrorestriction pattern (33). Notably, multiple copies of IS*Apl1* have previously been reported for some strains of A. pleuropneumoniae, in which IS*Apl1* can prevent ApxIV-based serological detection of serotype 7 strain AP76 due to the disruption of the *apxIV* gene (13). In our study, an analysis revealed that in addition to IS*Apl1*, 27 additional IS elements were located in 81 different positions in the serial *mcr-1*-containing isolates (see Table S1 in the supplemental material). Notably, despite this assortment of IS elements, only IS*Apl1* demonstrated a variable copy number in successive isolates, suggesting that this element is highly active. The patient was not undergoing any antibiotic treatment during his hospitalization, and the isolate was deemed to have colonized rather than infected the patient; thus, no obvious additional stresses were evident.

It is notable that within 2 weeks of the patient's repatriation to the United States, a second, unrelated E. coli isolate lacking *mcr-1* (and IS*Apl1*) was cultured from rectal and groin surveillance swabs. This ST-32 (clonal complex 32; phylogenetic group D) isolate eventually supplanted the *mcr-1*-containing strain, and the patient was colonized by the same strain 9 months later. Furthermore, the isolate remained sensitive to colistin and did not acquire the plasmid carrying *mcr-1*. Hence, in addition to the suspected fitness cost incurred by MCR-1-mediated modification of the lipopolysaccharide (5), high activity of IS*Apl1* may also play a role in reducing the fitness of *mcr-1*-harboring isolates. Further studies should help to clarify the role that this intriguing IS element plays in *mcr-1* mobilization and dissemination.

## MATERIALS AND METHODS

This report is the result of an infection control, quality improvement initiative authorized by policy memoranda #09-050 dated 25 June 2009 and #11-035 dated 23 April 2011. Additional research was approved by the institutional review board of the Walter Reed Army Institute of Research protocol number 1812 (version 6).

### Bacterial isolates and antibiotic susceptibility testing.

Strain identifications and antibiotic susceptibilities were determined using a BD Phoenix system (BD Diagnostics Systems, MD) with NMIC/ID 133 panels (Gram negative) in a College of American Pathologists (CAP)-accredited laboratory. MICs of colistin were determined using a broth microdilution method as previously described (34).

### Identification of *mcr-1* by real-time PCR.

A probe-based real-time PCR (RT-PCR) assay for the detection of *mcr-1* was performed with primers mcr-1-286F (5′-ACTTATGGCACGGTCTATGA-3′) and mcr-1-401R (5′-ACACCCAAACCAATGATACG-3′) and the HEX-labeled internal probe mcr-1-339Pr (5′-HEX-CCAAGCCGA-ZEN-GACCAAGGATC-3IABkFQ-3′). Sensitivity and specificity were determined according to minimum information for publication of quantitative real-time PCR experiments (MIQE) guidelines (35). Real-time PCR was performed in 20-μl volumes with 200 nM (each) primers and iQ Multiplex Powermix (Bio-Rad) on a Bio-Rad CFX96 real-time PCR instrument (Bio-Rad Laboratories, Hercules, CA). Cycling parameters were 95°C for 5 min and 40 cycles of 95°C for 10 s and 56°C for 40 s. Appropriate positive (E. coli MRSN 388634), negative (E. coli ATCC 25922), and no-template (water) controls were incorporated into every test. A 6-carboxyfluorescein (FAM)-labeled internal control targeting the 16S rRNA gene was incorporated into every reaction mixture as previously described (36).

### Long- and short-read whole-genome sequencing.

Short-read (550-bp) sequencing was performed using an Illumina MiSeq desktop sequencer (Illumina Inc., CA) as previously described (37). Newbler, version 2.7 (454 Life Sciences, CT), was used to assemble Miseq sequencing reads into *de novo* contigs and sequencing reads against reference DNA sequences. Single-molecule real-time (SMRT) sequencing was performed using a PacBio RS II instrument (Pacific Biosciences, CA), and sequencing reads were assembled *de novo* using HGAP 2.0 in the SMRT Analysis Portal. Comparative genomic analyses were performed using Geneious (Biomatters, Auckland, New Zealand). Antimicrobial resistance genes were annotated using ResFinder 2.1, transposon and insertion sequences were identified using ISfinder, and *in silico* plasmid replicon typing was performed using PlasmidFinder 1.3. Plasmid maps were generated using Easyfig 2.1.

### Accession number(s).

Complete genome sequences of all four *mcr-1*-containing isolates and their attendant plasmids have been deposited in the NCBI nucleotide database under accession numbers CP018103 to CP018126. The complete genome sequence of MRSN 346647, a representative of the second, unrelated, *mcr-1*-negative ST-32 E. coli strain, has been deposited under NCBI accession numbers CP018206 to CP018208. The sequence of the novel IS*5*-like element IS*Ec68* has been deposited at ISfinder and assigned accession number WP_000082736.1 at NCBI.

## Supplementary Material

Supplemental material
