# Supplementary material for: Analysis of Serial Isolates of mcr-1-Positive Escherichia coli Reveals a Highly Active ISApl1 Transposon
Source: Antimicrob Agents Chemother. 2017 Apr 24;61(5):e00056-17. doi: 10.1128/AAC.00056-17 (PMC5404521; doi:10.1128/AAC.00056-17)
Supplement: Supplemental material [file supp_61_5_e00056-17__index.html]

Analysis of Serial Isolates of mcr-1-Positive Escherichia coli Reveals a Highly Active ISApl1 Transposon — Supplemental material 

# Analysis of Serial Isolates of *mcr-1*-Positive Escherichia coli Reveals a Highly Active IS*Apl1* Transposon

## Supplemental material

- Supplemental file 1 -

  Table S1

  PDF, 17K
