# Supplementary material for: Analysis of Serial Isolates of mcr-1-Positive Escherichia coli Reveals a Highly Active ISApl1 Transposon
Source: Antimicrob Agents Chemother. 2017 Apr 24;61(5):e00056-17. doi: 10.1128/AAC.00056-17 (PMC5404521; doi:10.1128/AAC.00056-17)
Supplement: Supplemental material [file AAC.00056-17_zac005176126s1.pdf]

Supplemental Table S1. IS elements in MRSN 346355

IS[1]	# of copies[2]	Location[3]
1397	2	Chromosome
150	2	Chromosome
1A	10	Chromosome and plasmid
1F	3	Chromosome and plasmid
1R	4	Chromosome and plasmid
1X2	1	Chromosome
1X4	2	Plasmid
200C	4	Chromosome and plasmid
26	7	Chromosome and plasmid
3	4	Chromosome and plasmid
30	1	Chromosome
421	3	Chromosome
609	2	Chromosome
629	7	Chromosome
903B	6	Chromosome
Apl1	**4**	**Chromosome and plasmid**
Ec1	3	Chromosome
Ec12	2	Chromosome
Ec22	1	Chromosome
Ec23	2	Chromosome
Ec26	1	Chromosome

Ec38	1	Chromosome
Ec52	1	Chromosome
Ec68	1	Chromosome
Ec8	1	Chromosome
Kox3	3	Chromosome
Kpn26	2	Plasmid
Vsa3	1	Plasmid

[1] IS annotation was assigned based on full length matches from the ISFinder database (https://www-is.biotoul.fr/). IS*Apl1* is highlighted in bold, and is the only IS that shows variable copy number between the four serial *E.coli* isolates (See text for details)

[2] Total copy number found throughout the genome based on long-read sequence analysis.

[3] Location of IS element based on long-read sequence.
